# Apical root canal transportation of different pathfinding systems and their effects on shaping ability of ProTaper Next

**DOI:** 10.4317/jced.52309

**Published:** 2015-07-01

**Authors:** Sevinç-Aktemur Türker, Emel Uzunoğlu

**Affiliations:** 1 DDS, PhD, Department of Endodontics, Faculty of Dentistry, Bülent Ecevit University, Zonguldak, Turkey; 2 DDS, PhD, Department of Endodontics, Faculty of Dentistry, Hacettepe University, Ankara, Turkey

## Abstract

**Background:**

This study aimed to compare glide path preparation of different pathfinding systems and their effects on the apical transportation of ProTaper Next (Dentsply Maillefer, Ballaigues, Switzerland) in mesial root canals of extracted human mandibular molars, using digital subtraction radiography.

**Material and Methods:**

The mesial canals of 40 mandibular first molars (with curvature angles between 25° and 35°) were selected for this study. The specimens were divided randomly into 4 groups with 10 canals each. Glide paths were created in group 1 with #10, #15 and #20 K-type (Dentsply Maillefer, Ballaigues, Switzerland) stainless steel manual files; in group 2 with Path-File (Dentsply Maillefer) #1, #2, and #3 and in group 3 with #16 ProGlider (Dentsply Maillefer) rotary instruments; in group 4 no glide paths were created. All canals were instrumented up to ProTaper Next X2 to the working length. A double digital radiograph technique was used, pre and post-instrumentation, to assess whether apical transportation and/or aberration in root canal morphology occurred. Instrument failures were also recorded. The data were analyzed statistically using ANOVA and Tukey tests (p<0.05).

**Results:**

No significant differences were found among groups regarding apical transportation (*p*>0.05). Two ProTaper Next instruments failed in-group 4.

**Conclusions:**

Within the parameters of this study, there was no difference between the performance of path-finding files and ProTaper Next system maintained root canal curvature well and was safe to use either with path-finding files or alone.

** Key words:**Glide path, PathFile, ProGlider, ProTaper Next, transportation.

## Introduction

The introduction of nickel-titanium (NiTi) instruments allowed a safer and easier preparation of root canals with complex anatomic characteristics (1). However, during preparation, especially when preparing curved canals, iatrogenic errors, such as ledges, zips, perforations, and root canal transportation, can occur (2). Therefore, coronal enlargement and the prior creation of glide paths have been shown to minimize procedural errors during root canal treatment (3,4).

NiTi rotary instruments have been recently introduced on the market for the purpose of creating an initial glide path and eliminating the need for previous manual instrumentation. NiTi rotary mechanical preflaring was firstly introduced with the PathFile system by Dentsply Maillefer. The PathFile (PF; Dentsply Maillefer) NiTi pathfinding rotary system is manufactured from a conventional austenite NiTi alloy for canal preflaring. The system consists of three instruments with ISO 13, 16, 19 tip sizes with a .02 taper (3). The new ProGlider (PG) NiTi Rotary instrument for mechanical preflaring was recently introduced by Dentsply (Maillefer, Ballaigues, Switzerland). It is manufactured using M-Wire NiTi alloy to enhance flexibility and cyclic fatigue resistance as claimed by the manufacturer. The system consists of a single instrument, with a variable progressive taper. The PG instrument is available in 21, 25 and 31 mm length and tip size 16 with a taper of .02 (5).

The aim of the present study was to compare glide path preparation of ProGlider, PathFile and K-type files and their effects on the apical transportation of ProTaper Next in the occurrence of apical transportation in curved root canals.

## Material and Methods

Forty human mandibular first molars with curved mesial canals extracted for periodontal reasons were used. The curvature angle of the canals was determined according to the method described by Schäfer *et al.* (6) Canals with curvatures between 25°and 35° were included in the study. The mesiobuccal root was used to investigate transportation. The crown and distal root of each tooth were removed at the level of the cemento-enamel to obtain root canal measuring 12 mm in length for specimen standardization. Apical patency was confirmed with a number # 08 stainless steel manual K-type file. To avoid any bias caused by differences in the initial width, all the canals that, before any instrumentation, could be easily negotiated up to the apex with a #15 (or wider) file were not included in the study. The working length (WL) was determined by subtracting 1 mm from the length measured when the tip of the file was first observed at the apical foramen.

Keeping the #10 K file inside the canal, a series of radiographs were taken before instrumentation. To ensure consistent radiographs for all specimens, an L-shaped wooden platform was manufactured to position the head of the x-ray tube perpendicular to the digital sensor at a focal distance of 30 cm. The acrylic jig containing the root was then positioned at the center of the sensor so as to align perfectly with a square-shaped guide previously designed on the sensor, thus allowing the jig to be accurately repositioned during the experimental procedure. After sealing the apical foramen using a small piece of wax, roots were embedded in 2 x 2 x 2 mm acrylic resin blocks so that they could be removed for preparation and later reinserted in a predetermined position for the purpose of comparing the images taken before and after preparation using standardized radiographic imaging. Adobe Photoshop CC 2014 software (Adobe Systems Inc, San Jose, CA) was used to enhance the edges of the initial and final instrumentation radiographs (4).

Roots were randomly divided into 4 experimental groups (n=10):

Group 1: (M) Canals preflared with #10, #15, and #20 stainless steel manual K-type files and instrumented using the ProTaper Next system.

Group 2: (PF) Canals preflared with Path-File #1, #2, and #3 and instrumented using the ProTaper Next system.

Group 3: (PG) canals preflared with #16/02, ProGlider instrument and instrumented using the ProTaper Next system.

Group 4: (PTN) Canals instrumented using ProTaper Next system without a previous glide path.

The rotary file systems were performed with an electrical motor (X-Smart, Dentsply Maillefer, Ballaigues, Switzerland) a 16:1 reduction hand-piece at 300 rpm. ProTaper Next files were used in the sequence X1 and X2 according to the manufacturer’s instructions. In all groups, the instruments were used up to their total WL. Each instrument was used in 3 canals and then discarded.

Irrigation was performed with 2 mL 2.5% NaOCl solution after each file change. 1 mL of 17% EDTA was applied for 3 minutes followed by final irrigation with distilled water.

-Evaluation of the Root Canal Preparation

After performing the preparations with path-finding systems, the roots were repositioned in predetermined position in the acrylic jig, and postoperative radiographs were taken with a #15 stainless steel manual file inside the canal. After creating glide paths, canals further prepared with ProTaper Next system up to X2, and again postoperative radiographs were taken with a PTN X2 rotary file inside the canal in predetermined position in the acrylic jig.

Briefly, images were obtained at 3 times in-groups 1, 2 and 3; with 10 K-file, 15 K-file and PTN X2 file placed inside the root canal and adjusted to the WL. Images were obtained at 2 times in-group 4 with ≠10 K-files and PTN X2 file (no glide paths were created).

**Figure 1:**
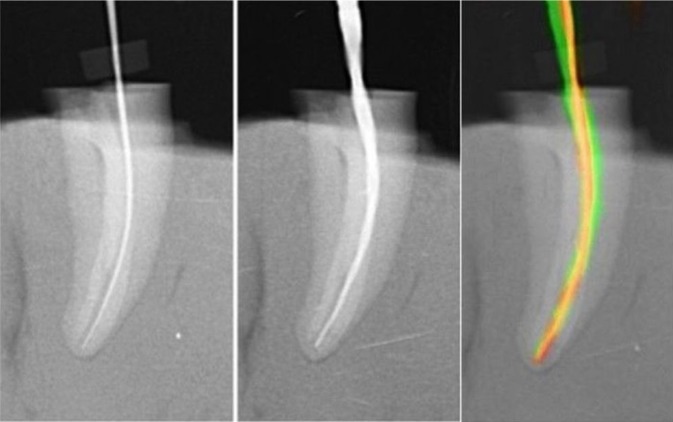
Representative pre-operative, post-operative and superimposed pre- and post- operative images of curved root canals prepared with ProTaper Next.

The digital radiographs were taken in the same apparatus for the whole sample and were saved in JPG format and imported into Adobe Photoshop CC 2014 software. The images of the pre- and post-instrumentation radiographs were then superimposed to compare the differences between pre- and post-instrumentation canal geometry (Fig. 1). The superimposed images were then transferred to Image J software (Image-J v1.44; US National Institutes of Health, Bethesda, MD, USA). The distance between the pre and post instrumentation file tips at the WL was measured, and this measurement was assumed as the extent of the apical transportation (in millimeters). One operator completed all root canal preparations whilst a second examiner who was blind in respect of all experimental groups carried out the assessments of the apical transportation both after path-finding files and ProTaper Next files. The data were distributed normally (Shapiro-Wilk test) and were analyzed statistically using the analysis of variance (ANOVA) and post-hoc Tukey-test at a significance level of *p* < 0.05.

## Results

**Figure 2:**
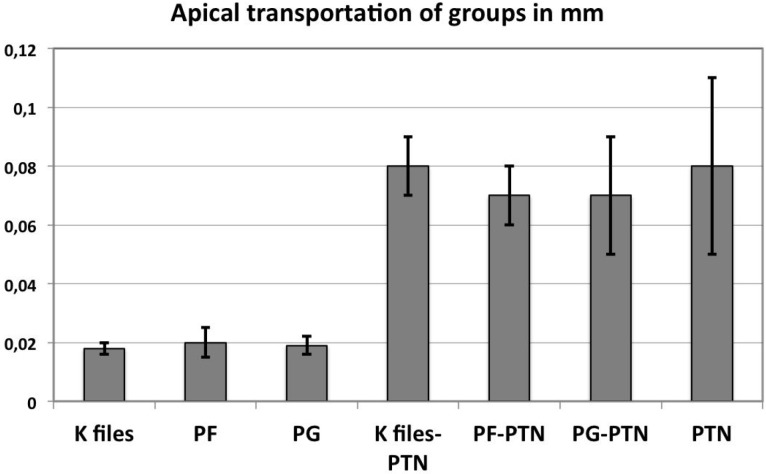
Mean transportation of curved canals (mm) and SD after canal preparation with the different instruments (n = 10 canals in each group).

Two teeth from the PTN group (without glide path group) were lost as a result of instrument fracture. One of the fractured instru-ments was PTN X1 and the other was PTN X2. However, these teeth were replaced. Fig. 2 showed mean transportation of curved canals (mm) and SD after canal preparation with the different instruments. There were no significant differences between the path-finding files regarding apical transportation values (*p* = 0.329). Further preparation with ProTaper Next was resulted in increasing apical transportation values (Fig. 2). The highest apical transportation measurement was done in group 4 (without a previous glide path preparation). However, there were no significant differences among groups (previously prepared with path-finding files or not-prepared) afterwards ProTaper Next preparation, regarding apical transportation values (*p* = 0.215).

## Discussion

A number of procedural errors, such as apical transportation, ledges, changes in the angle of canal curvature, may occur during the shaping of curved canals (7). Creating a glide path has proven to be essential for allowing the safer use of NiTi rotary instrumentation (3). Several methods have been used to investigate the efficiency of the instruments and techniques for root canal preparation. Amongst these digital radiographic imaging method is commonly used to compare canal shape before and after instrumentation (4,8,9). Digital subtraction radiograph is obtained by eliminating anatomical structures on a radiographic image by digitally storing baseline and post-treatment images and then combining them together to display the final subtracted image, which emphasizes the differences between the two original films (10).

The present study aimed to compare the new ProGlider path-finding file with K-file and PathFile and their effect on the shaping ability of ProTaper Next system using digital subtraction radiography. The present study results showed that the instruments tested did not differ with respect to apical transportation. After preparation, the major part of the foramens kept their same initial preparation position, and the shape of the prepared canals maintained the same central axes that existed before the preparations. These results were in accordance with some previous reports. Alves *et al.* (4) has shown that PathFile rotary instruments used alone (without a subsequent instrument) did not have any influence on apical transportation or canal aberration when compared with K-type stainless steel manual files. Similarly, D’Amario *et al.* (8) found no differences between K-type files and PathFile in apical transportation. In contrast with the present study, Berutti *et al.* (3) and Pasqualini *et al.* (11) suggested that NiTi rotary PathFile instruments preserve the original canal anatomy, cause less canal aberrations when compared with K-files.

According to the results of present study regarding apical transportation, there were no statistically significant differences between the groups prepared with ProTaper Next either combined with path-finding files or not. In a previous study Zanette *et al.* (12) found no differences between apical transportation after instrumentation with ProTaper Universal F3 or F4 files used with and without glide path.

ProGlider file has been newly introduced to market. Therefore, there are limited reports concerning its shaping ability. In a recent publication (5), it has been reported that ProGlider instrument had a significantly higher flexibility, higher resistance to cyclic fatigue and torsional stress than PathFile instruments. Moreover, in a CBCT study, Elnaghy and Elsaka (13) reported that creating a glide path with ProGlider revealed better performance with fewer canal aberrations when compared with instrumentation performed with ProTaper Next with PathFile or ProTaper Next only. The differences between two studies may be due to the discrepancies in the study designs. In the present study the apical transportation was evaluated only at 1 mm from the apex by using digital substraction radiography. However, Elnaghy & Elsaka (13) evaluated the apical transportation at 3, 5 and 7 mm from the apex by using cone-beam computed tomography imaging.

It has been concluded that apical transportation that are more than 0.3 mm can jeopardize the outcome of treatment due to a significant decrease in the sealing ability of root filling material (14,15). None of the transportation values measured in this study surpassed this limit. ProTaper Next instruments are made from M- wire. These instruments are characterized by an innovative off-centered rectangular cross section that is claimed to give the files a snake-like swaggering movement as it advances into the root canal. Also the manufacturer recommends the creation of a glide path prior to canal preparation. The present apical transportation values calculated after ProTaper Next preparation corroborate with previous studies (16,17).

## Conclusions

In conclusion, there was no difference between the performance of path-finding files regarding apical transportation and ProTaper Next system maintained root canal curvature well and was safe to use either with path-finding files or without them.
